# Epidemiologic Characteristics of Adolescents with COVID-19 Disease with Acute Hypoxemic Respiratory Failure

**DOI:** 10.1155/2022/7601185

**Published:** 2022-09-05

**Authors:** Helen Kest, Ashlesha Kaushik, Somia Shaheen, William Debruin, Sahil Zaveri, Mario Colletti, Sandeep Gupta

**Affiliations:** ^1^Division of Pediatric Infectious Disease, Department of Pediatrics, St. Joseph's Children's Hospital, Paterson 07503, NJ, USA; ^2^Pediatric Infectious Diseases, Unity Point Health at St. Luke's Regional Medical Center and University of Iowa Carver College of Medicine, Sioux 51104, IA, USA; ^3^Department of Pediatrics, St. Joseph's Children's Hospital, Paterson 07503, NJ, USA; ^4^Pediatric Intensive Care, Department of Pediatrics, St. Joseph's Children's Hospital, Paterson 07503, NJ, USA; ^5^St. Joseph's Health, Paterson 07503, NJ, USA; ^6^Pediatric Intensive Care, Department of Pediatrics, St. Joseph's Children's Hospital, Paterson 07503, NJ, USA; ^7^Pulmonary and Critical Care, Unity Point Health at St. Luke's Regional Medical Center, Sioux 51104, IA, USA

## Abstract

We report our experience of COVID-19 disease with hypoxemic respiratory failure among patients aged 12–21 years admitted to the intensive care unit at two tertiary care institutions in Northeastern and Midwestern United States. Our results showed that during the main study period that spanned the initial surge at both geographic locations, adolescents with SARS-COV-2 infection admitted to the ICU with respiratory failure were more likely to be male, black, and morbidly obese and with two or more comorbidities. The majority (79%) were admitted with COVID-19-related pneumonia and 15 developed respiratory failure; two-thirds of patients with respiratory failure (9/15, 60%) required mechanical ventilation (MV). More than two-thirds of patients (11/15, 75%) with respiratory failure were obese with BMI > 30 compared to those without respiratory failure (*p* < 0.0001), and those with BMI > 40 were 4.3 times more likely to develop respiratory failure than those with normal BMI; 40% of patients with respiratory failure had two or more pre-existing medical comorbidities. Inflammatory markers were 2–20 times higher in patients with respiratory failure (*p* < 0.05). The majority of patients on MV (7/9) developed complications, including ARDS (acute respiratory distress syndrome), acute renal injury, and cerebral anoxic encephalopathy. Patients with respiratory failure had a significantly longer length of hospital stay than patients without respiratory failure (*p* < 0.05). The majority of the admitted adolescents in the ICU received steroid treatment. None of the patients died. An additional review of a 6-month postvaccination approval period indicated that the majority of ICU admissions were unvaccinated, obese, black patients and all patients who developed respiratory failure were unvaccinated. Our study highlights and supports the need for maximizing opportunities to address vaccination and healthcare gaps in adolescents as well as promoting public health measures including correct use of masks, effective vaccination campaigns for this age group, and additional passive preventive interventions for COVID-19 disease in adolescents especially with comorbid conditions, and in minority populations.

## 1. Introduction

Globally, COVID-19 disease caused by SARS-COV-2 continues unabated with over 400,000, 000 confirmed cases including about 6 million deaths, reported to WHO as of March 2, 2022 [1]. In the United States, more than 79 million cases and approximately 900,000 COVID-19-associated deaths have occurred during the same period [2]. While children and adolescents have contributed to the global burden, the disease has been mostly mild in this age group. Adolescents as a group are more likely to have adult-like complications and more likely to develop severe illnesses [3].

Severe COVID-19 disease is caused by unabated viral replication in pneumocytes and alveolar epithelial cells, with resulting exaggerated inflammatory immune response that leads to causing alveolar damage and vascular leakage [4, 5]. Management of hypoxemic respiratory failure has evolved over the years with the utilization of HFNC for selected patients as an alternative to invasive mechanical ventilation. High-flow nasal cannula (HFNC) oxygen therapy is a recent technique that delivers a high flow of heated and humidified gas at flow rates of up to 60 liters/min, which can be titrated, based on the patient's flow and fi02 requirements [6–9].

Here, we characterize adolescent patients with severe Covid-19 respiratory disease who were admitted to two ICU centers in NE and MW US.

## 2. Methods/Study Design

The objective of our retrospective descriptive study was to define the epidemiologic characteristics of adolescent patients with COVID-19 respiratory failure admitted to Intensive care units (ICUs) at two tertiary care centers in Northeastern and Midwestern United States (NJ and Iowa) during the initial wave of the COVID-19 pandemic. Board-certified intensivists run both ICUs and both ICUs are in locations where climate, COVID epidemiologic rates, and bed sizes are similar.

Adolescence was defined as the age group 12–21 years of age, per WHO definition. All adolescents admitted to the ICUs with positive SARS-COV-2 PCR during the study period at the two tertiary care centers were included. The main period of study occurred during our initial surge (March 14, 2020, to October 31, 2020). Pfizer-BioNTech COVID-19 vaccine received the U.S. Food and Drug Administration (FDA) Emergency use authorization for adolescents aged 16–17 years old on December 11, 2020, and for children aged 12–15 years old on May 10, 2021. We conducted additional subgroup analysis on this cohort following approval (June 1 to December 31, 2021). Patients with other viral/bacterial coinfections were excluded. Patients with hypoxemic respiratory failure were compared with those without respiratory failure. Respiratory failure was defined based on the use of a high-flow nasal cannula or mechanical ventilation. The use of respiratory support was at the discretion of the ICU provider. Patients with PaO_2_/FiO_2_ ratio < 300 who are unable to maintain their O_2_ saturation above 92% with a nonrebreathing mask were started on HFNC or were intubated depending on their ability to ventilate as per ICU clinical decision making.

We reviewed medical records of patients hospitalized with SARS-COV-2 in intensive care units at our tertiary health care systems in Northeastern NJ and Northwestern IA, the United States to describe trends and patterns and respiratory complications. Pre-existing chronic conditions were analyzed by the Agency for Healthcare Research and Quality Chronic Condition Indicator tool [10] using ICD-10-CM diagnoses.

Testing for SARS-CoV-2 on nasopharyngeal swabs was done by use of real-time reverse transcription polymerase chain reaction (rt-PCR; BioGX SARS-CoV-2 BD MAX™ kit (Becton, Dickinson, MD, USA, and Xpert® Xpress SARS-CoV-2 kit (Cepheid, Sunnyvale, CA, USA) during initial hospital admission. This study was approved by our respective institutional review boards as exempt from consent. Comparisons between study groups for categorical variables were performed with chi-square [2] or Fisher's exact test. GraphPad Prism version 8 (GraphPad Software, San Diego, CA) was used to analyze the data. We calculated adjusted odds ratios (AORs) and 95% CIs for respiratory failure for sex, race/ethnicity, and presence of chronic conditions. All hypothesis testing was 2-sided and a *p* value of < .05 was considered statistically significant.

## 3. Results

A total of 47 adolescents were admitted to the intensive care unit during the main study period at both geographic locations (38 in NJ and 9 in Iowa). Demographic and clinical characteristics of all hospitalized patients at both geographic locations are presented in Table 1.

The majority of adolescents (79%) were admitted with COVID-19-related pneumonia and a third of the total admitted 15 (32%) developed respiratory failure. Two patients were admitted with COVID-19 triggered Diabetic ketoacidosis. None of the patients were diagnosed with myocarditis or multisystem inflammatory syndrome in children (MIS-C).

The median age was 13 years (IQR 12–18 years) and 57% of patients were female (male: female ratio 1:1.3). A significantly greater proportion (32/47, 68%) of adolescents needing ICU admission were Black non-Hispanic or Latino Hispanic (*p* < 0.05). Fever (35%) and respiratory symptoms 61.6%) were the common presenting symptoms followed by GI symptoms (27.6%) in form of nausea and vomiting. A majority (9/11) of pts presenting with shortness of breath developed respiratory failure (*p*=0.0002).

The majority of obese patients (87% (11/13)) admitted to ICU developed respiratory failure and all patients (6/6, 100%) with 2 or more comorbidities (including diabetes mellitus type 1, cerebral palsy, Prader Willi syndrome, asthma) developed respiratory failure (*p* < 0.0001).

Chest radiographs showed bilateral infiltrates in 11(23%) patients. Eighty-nine percent (42/47) of all ICU admissions received COVID-19 therapeutics. The majority (26/47, 55.3%) of patients in the ICU received steroids for treatment; 8 (17%) patients received hydroxychloroquine; 5 patients received (10.6%) remdesivir; 3 received azithromycin (6.4%); 1 (2.1%) received tocilizumab. One-third of the patients (30%) received empiric antibiotics for ≤ 48 hours, and 6 patients (12.7%) needed inotropic support. Most (89%) of the patients were discharged at ≤ 14 days. All patients that did not require HFNC or mechanical ventilation were discharged at ≤ 14 days compared to only a third of those with respiratory failure (33%, 32 *vs*. 5, *p* < 0.0001).  Table 2. Selected treatment and outcome characteristics of hospitalized patients.

### 3.1. Respiratory Failure

About one-third of all adolescents admitted to the ICU had respiratory failure (15/47, 32%).

A significantly higher proportion of black patients compared to other ethnic groups admitted to the ICU developed respiratory failure (57%) (46% whites and 16% Latino, *p* < 0.01). All patients with respiratory failure presented with respiratory symptoms at admission. A high proportion of patients with respiratory failure (6/15, 40%) had two or more pre-existing medical conditions/comorbidities, and more than two-thirds of patients (11/15, 75%) with respiratory failure were obese with BMI > 30 compared to those without respiratory failure (*p* < 0.0001). Patients with BMI > 40 were 4.3 times more likely to develop respiratory failure than those with normal BMI.

Inflammatory markers (LDH, CRP, CPK, IL-6) were 2–20 times higher among patients with respiratory failure compared to patients without respiratory failure (*p* < 0.05). IL-6 was only obtained in two patients. Median CRP levels were elevated three times (58.15 *vs*. 20.8), median LDH levels were elevated twice (358 *vs*. 158.5), median CPK 14 times (387.5 *vs*. 27.5), and median IL-6 levels were elevated 20 times in patients with respiratory failure than patients without respiratory failure ((162.1 *vs*. 6.4), (*p* < 0.05)).  Table 3 shows selected laboratory characteristics of hospitalized patients.

All patients with respiratory failure (15/15) received COVID-19 therapeutics compared to 19 (59%) patients without respiratory failure (*p* < 0.0001). All patients with respiratory failure received steroids and 5/15 (33%) patients with respiratory failure received remdesivir compared to none of the patients without respiratory failure (*p*=0.002). The mean duration of hospitalization was significantly longer for those with respiratory failure (16.9) (±2.8) days versus 10.3 ± (3.5) days, (*p* < 0.001). Three patients stayed for more than 4 weeks, compared to none of the patients without respiratory failure (*p* < 0.0001).

Among the 15 patients that required respiratory support, 9 (60%) required invasive mechanical ventilation. All (100%) of those requiring MV had underlying asthma. The mean duration of mechanical ventilation was 11 (±1.6) days. Complications were seen in 7/9 (77%) of the mechanically ventilated patients, including ARDS (acute respiratory distress syndrome), acute renal injury, and one patient who sustained cerebral anoxic encephalopathy. None of the patients died.

### 3.2. Subgroup Analysis

Analysis of a subgroup of comparison patients admitted from June 1, 2021, to December 21, 2021, showed 22 adolescents admitted to intensive care units at both geographic locations (12 in NJ and 10 in Iowa).  Table 3, subgroup analysis, shows the demographic and clinical characteristics of all hospitalized patients at both geographic locations. The pattern was similar: a majority of adolescents (75%) were admitted with COVID-19-related pneumonia and a third (32%) developed respiratory failure. 95% (21/22) of adolescents were unvaccinated and all patients with respiratory failure were unvaccinated. All patients with respiratory failure were unvaccinated and obese; a majority (4/7, 57%) were black, one had multiple comorbid factors-obesity (BMI > 40), OSA, and SCD. Among the seven patients with respiratory failure, 3 (43%) required invasive mechanical ventilation. All (100%) of those requiring MV had underlying asthma and obesity. The mean duration of mechanical ventilation was 8 days. All patients that did not require HFNC or mechanical ventilation were discharged at ≤ 7 days.

Initial data from the subgroup shows the emergence of a new picture as compared with the main period. ICU admissions and respiratory failure occurred mostly in unvaccinated, obese, and minority (mostly black) adolescents.

## 4. Discussion

Our study describes the epidemiology of severe SARS-COV-2 infection based on the presence or absence of respiratory failure during the period March 2020 to October 2020 in the adolescent population at two different geographic locations in the Northeastern and Midwestern United States. Additionally, we conducted a subgroup analysis of patients admitted to both centers following vaccination approval and availability for patients ≥ 12 years old [11].

More than 75% of adolescents in our study were admitted with pneumonia, and a large proportion developed respiratory failure. Overall, a greater proportion of black African American patients developed respiratory failure. Morbid obesity posed a significant risk factor for developing respiratory failure. Two or more comorbidities including diabetes mellitus and pre-existing respiratory disease also were significant risk factors for developing respiratory failure. Our study shows a correlation between body habitus, and other comorbid factors that affect respiratory function placing older children and adolescents at high risk for complicated COVID-19 with respiratory failure. Current data, from the 2017–2018 National Health and Nutrition Examination Survey (NHANES), shows that 19.3% of U.S. children and adolescents are obese with the adolescent age group accounting for the highest rates at 21.2% [12]. Our study highlights the importance of interventions directed at curbing obesity in adolescents.

While adolescents may represent a small fraction of SARS-COV-2 hospitalization, significant illnesses needing ICU care can occur. Adolescents in our study were severely affected and a large proportion of those with respiratory failure needed MV.

Inflammatory markers like IL-6 and CPK were > 15 times elevated in the respiratory failure group. Patients with respiratory failure had significantly higher values of inflammatory markers portending a poor prognosis and the majority of patients with MV had complications. None of our patients died. Utilization of available COVID-19 therapeutics was greater in the respiratory failure group according to NIH guidelines at the time, and steroids were most commonly used. Additional analysis was done after availability of vaccines and showed that the majority of adolescents that were admitted to the ICU and all of those developing respiratory failure were unvaccinated and obese. Our results thus show that there is a need for encouraging the vaccination of adolescents at all levels. Additionally, other preventive measures including management of obesity as mentioned above, as well as, consideration for passive immunoprophylaxis need to be encouraged as well.

The medical community has been radically changed in terms of pandemic possibilities and the potential for new threatening trends. Adults typically bear the weight of most trends. Early prevention intervention in adolescents may be the next great move to curb disease complications.

## 5. Conclusions

Adolescents during the initial wave were severely affected, and a large proportion had respiratory failure and needed MV. Evaluation of recent trends shows that unvaccinated minority adolescents who are obese remain at risk for respiratory support and would benefit from targeted efforts to promote vaccinations. Additionally, this group should be considered for COVID-19 passive immune therapy and therapeutics to prevent complications. As COVID-19 rages on with its changing epidemiology and variants surge all across the globe, preventive interventions in adolescent health would play a central role in promoting adolescent as well as adult health (See Table 4).

## Figures and Tables

**Table 1:** Demographic and clinical characteristics of hospitalized patients.

Characteristics	Total (*n* = 47)
Age, *y*, median (IQR)	13 (12–18)

Sex	
Female, *n* (%)	27 (57%)
Male, *n* (%)	20 (43%)

Race	
Black, *n* (%)	7 (13%)
White, *n* (%)	15 (32%)
Latino, *n* (%)	25 (40%)

Obesity (BMI > 30), *n* (%)	13 (27.6%)

Comorbidities	
Respiratory, *n* (%)	9 (19.1%)
Hematologic/malignancy/immunosuppression, *n* (%)	2 (4.2%)
Diabetes/prediabetes/endocrine	5 (10.6%)
Neurologic/psychiatric, *n* (%)	11 (23.4%)
2 or more comorbidities, *n* (%)	6 (12.7%)

Presenting symptoms/history (*n* = 47)	
Cough, *n* (%)	16 (34%)
Fever, *n* (%)	21 (45%)
Shortness of breath, *n* (%)	11 (23.4%)
Headache, *n* (%)	3 (6.4%)
Rhinorrhea, *n* (%)	2 (4.2%)
Nausea/vomiting, *n* (%)	9 (19.1%)
Diarrhea, *n* (%)	4 (8.5%)
Myalgias, *n* (%)	2 (4.2%)

Admission diagnosis (*n* = 47)	
Respiratory failure *n* (%)	15 (32%)
Shock, *n* (%)	3 (6.4%)
Pneumonia, *n* (%)	37 (79%)
Dka, *n* (%)	2 (4.2%)
Seizures, *n* (%)	5 (13.2%)

**Table 2:** Selected treatment and outcome characteristics of hospitalized patients.

Characteristics	Total (*n* = 47)
COVID-19 therapy	
Hydroxychloroquine, *n* (%)	8 (17%)
Azithromycin, *n* (%)	3 (6.4%)
Remdesivir, *n* (%)	5 (10.6%)
Corticosteroid, *n* (%)	26 (55.3%)
IL-6 inhibitor, *n* (%)	1 (2.1%)

Complications	
ARDS, *n* (%)	4 (8.5%)
AKI *n* (%)	2 (4.2%)

Anoxic cerebral encephalopathy	1 (2.1%)

Outcomes	
Status on hospital day 14, *n* (%)	
Discharged (%)	42 (89%)
Mortality, *n* (%)	0 (0%)

**Table 3:** Selected laboratory characteristics of hospitalized patients.

Characteristics	Total (*n* = 47)
WBC, k/*µ*L, median (IQR) (*n* = 47)	9.8 (7.5–13.7)
Hemoglobin, g/dL, median (IQR) (*n* = 47)	13.3 (12.1–14.6)
Platelets, k/*µ*L, median (IQR) (*n* = 47)	270 (230–354)
AST, U/L, median (IQR) (*n* = 47)	24 (18.25–34.75)
ALT, U/L, median (IQR) (*n* = 47)	18.5 (10.5–27.75)
Total bilirubin, mg/dL, median (IQR) (*n* = 47)	0.45 (0.4–0.7)
BUN, mg/dL, median (IQR) (*n* = 47)	10 (7–11)
Creatinine, mg/dL, median (IQR) (*n* = 47]	0.62 (0.38–0.81)
C-reactive protein, mg/dL, median (IQR) (*n* = 18)	43.75 (5.85–189.05)
Procalcitonin, ng/mL, median (IQR) (*n* = 10)	0.125 (0.0575–4.67)
Pro-BNP, pg/mL, median (IQR) (*n* = 9)	54 (19–396)
Troponin, ng/mL, median (IQR) (*n* = 13)	0.011 (0.01–0.048)
CPK, U/L, median (IQR) (*n* = 11)	62.5 (27.25–225.75)
Lactate, mmol/L, median (IQR) (*n* = 13)	1 (0.9–2.2)
D-dimer, *µ*g/mL FEU, median (IQR) (*n* = 17)	1.48 (0.6525–3.01)
LDH, U/L, median (IQR) (*n* = 17)	279 (157.25–341)
IL-6, pg/mL, median (IQR) (*n* = 2)	84.25 (45.325–123.175)

**Table 4:** Subgroup analysis: Characteristics of patients admitted postvaccination availability.

Characteristics	Total (*n* = 22)
Age, *y*, median (IQR)	16 (13–18)

Sex	
Female, *n* (%)	15 (68%)

Race	
Black, *n* (%)	9 (41%)
White, *n* (%)	7 (32%)
Latino, *n* (%)	6 (27%)

Obesity (BMI > 30), *n* (%)	14 (64%)

Comorbidities	
Respiratory, *n* (%)	9 (41%)
Hematologic/malignancy/immunosuppression, *n* (%)	4 (18%)
Diabetes/prediabetes/endocrine	3 (14%)
Neurologic/psychiatric, *n* (%)	2 (9%)
2 or more comorbidities, *n* (%)	5 (23%)

Outcomes	
Discharged (%)	12 (100%)
Mortality, *n* (%)	0 (0%)

## Data Availability

All data used in the study are included in the article.
